# Safety and Feasibility of a Two-Way Audiovisual Teleconferenced Pulmonary Rehabilitation Program

**DOI:** 10.1016/j.chpulm.2024.100089

**Published:** 2024-07-29

**Authors:** Emily S. Wan, Josephine Decherd, Christine Stella, Jonathan R. Venne, Brenda McKeon, Stephanie A. Robinson, Patricia Bamonti, Marilyn L. Moy

**Affiliations:** a Pulmonary & Critical Care Section, VA Boston Healthcare System, Boston, MA; b Channing Division of Network Medicine, Brigham & Women’s Hospital, Boston, MA; c Harvard Medical School, Boston, MA; d Center for Healthcare Organization and Implementation Research, VA Bedford Healthcare System, Bedford, MA; e The Pulmonary Center, Boston University School of Medicine, Boston, MA; f Research and Development, VA Boston Healthcare System, Boston, MA; g Department of Psychiatry, Harvard Medical School, Boston, MA

**Keywords:** COPD, directly supervised exercise, pulmonary rehabilitation, telerehabilitation, two-way audiovisual teleconferencing, virtual pulmonary rehabilitation

## Abstract

**Background:**

Given limited access to center-based, in-person pulmonary rehabilitation (PR), alternative delivery strategies are needed.

**Research Question:**

We compared a virtual PR program with a conventional center-based one with respect to safety, feasibility/acceptability, and geographic catchment (primary outcomes). We explored efficacy by examining changes in functional outcomes (secondary outcomes).

**Study Design and Methods:**

This single-center observational real-world report included patients enrolled from July 30, 2020, through June 30, 2023, who attended one or more PR class. Patients undergoing virtual PR exercised in their homes under direct supervision via two-way audiovisual teleconferencing. Baseline demographic information and adverse events were extracted from electronic medical records. Google Maps estimated distance and drive time from residential addresses to the PR center. Intake and exit evaluations for secondary (functional) outcomes and feedback questionnaires were completed in a subset.

**Results:**

A total of 120 (52 in-person and 68 virtual) patient enrollments were examined; 84% of patients had COPD. Mean age, FEV_1_ and FVC % predicted, and baseline 6-min walk test distance were similar between groups. For safety, the overall rate of PR-related adverse events was 1.2 per 1,000 person-days of observation, with no between-group differences. For feasibility, the average number of exercise classes completed (12.4 ± 6.2 vs 13.0 ± 6.1) and proportion of patients completing ≥ 70% of classes (61.5% vs 67.6%) was comparable between the in-person and virtual groups, respectively. For acceptability, among those who completed the virtual PR feedback questionnaire (n = 30), 100% felt safe exercising at home, 97% endorsed clear internet connection, and 90% agreed education sessions were easy to understand. For geographic catchment, patients in virtual PR lived farther (median, 34.1 miles; interquartile range, 16.6-45.1 vs median, 10.3 miles; interquartile range, 5.6-20.6 miles; *P* < .001) and had longer drive times (mean 86.0 ± 31.6 vs 51.4 ± 31.9 min; *P* < 0.001) than patients in in-person PR. In the subset with both intake and exit evaluations, similar improvements were observed in functional outcomes and dyspnea in both groups.

**Interpretation:**

This study suggests that two-way audiovisual teleconferenced PR is safe, feasible/acceptable, and significantly expands geographic catchment.

**Take-home Points**

**Study Question:** How does a virtual pulmonary rehabilitation program compare to a contemporaneous traditional center-based program with respect to safety, feasibility, and geographic catchment in a real-world setting?

**Results:** In this observational cohort, the total adverse event rate was low and pulmonary rehabilitation completion rates were comparable by modality; individuals enrolled in the virtual program lived significantly farther, with longer drive times.

**Interpretation:** Inclusion of a virtual option may improve access to pulmonary rehabilitation for individuals with chronic lung disease.

Pulmonary rehabilitation (PR) provides supervised aerobic exercise training, strength training, and multidisciplinary self-management education for people with chronic lung diseases, including COPD.^1^, ^2^, ^3^ Center-based (in-person) PR has been studied extensively and has been shown to improve exercise tolerance and health-related quality of life (HRQoL), reduce symptoms, and has been associated with decreased risk for hospitalizations and death in people with COPD.^4^^,^^5^ Center-based PR is cost-effective^6^ and is considered standard of care for symptomatic patients with COPD.^1^, ^2^, ^3^^,^^5^

Despite the benefits of PR, participation in conventional center-based programs remains limited, with only 1.5% to 3.7% of US Medicare and Veterans Health Administration beneficiaries using PR.^7^^,^^8^ Patients who are referred to center-based PR face significant barriers, including the need to travel long distances multiple times a week.^9^^,^^10^ Because most PR programs are located in urban areas, access for rural patients remains limited.^11^^,^^12^ Among Medicare beneficiaries, two-fifths of older adults with COPD had poor access to PR, with markedly worse access for patients in rural areas.^13^ With center-based programs at risk for closure due to inadequate reimbursement^14^ and disruptions from the COVID-19 pandemic, there is a need to identify alternative PR delivery strategies.

Telerehabilitation, defined as “the delivery of medical or rehabilitative care to persons with rehabilitation needs via telecommunication or the internet”^15^ holds promise to address the gap in PR access. Studies support that telerehabilitation for people with chronic respiratory disease achieves functional outcomes similar to those of center-based PR.^16^, ^17^, ^18^ However, in current telerehabilitation models, which have been developed mainly outside the United States, considerable heterogeneity with respect to asynchronous or unsupervised sessions, requirements for additional resources or staffing, or reliance on prerecorded or written educational materials exists.^16^^,^^19^, ^20^, ^21^ There is substantial variation in the degree to which virtual PR programs maintain fidelity to the essential components (eg, comprehensive intake and exit evaluations, individualized exercise prescriptions and progression plan, aerobic exercise, strength training, education) of conventional center-based PR programs.^2^^,^^5^^,^^16^^,^^22^ Finally, the impact of telerehabilitation on safety, patient engagement, and geographic catchment in real-world settings outside of research trials has been incompletely examined.^16^^,^^19^, ^20^, ^21^^,^^23^^,^^24^

The VA Boston center-based PR program, continuously certified by the American Association of Cardiovascular and Pulmonary Rehabilitation since 2013, leveraged existing infrastructure in 2020 to develop a synchronous virtual program in response to the COVID-19 pandemic. In this pragmatic, real-world report, we compare telerehabilitation via VA Video Connect (VVC), a secure two-way audiovisual communications technology platform, with conventional center-based in-person PR with respect to primary outcomes of safety, feasibility/acceptability, and geographic catchment. Secondary (exploratory) outcomes included changes in dyspnea and functional measures both within and between the two groups.

## Study Design and Methods

### Patients

This single-center observational cohort study included all patients enrolled in PR at the VA Boston Healthcare System from July 30, 2020, to June 30, 2023, who attended one or more PR class (inclusion criteria). Patients who attended PR classes using both face-to-face (F2F) and VVC modes during the same enrollment due to evolving COVID-19 policies were excluded from analysis (n = 3).

Patients completed intake and exit evaluations which included questionnaires, a 6-min walk test (6MWT), a 30-s sit-to-stand (STS) test, and the Timed Up and Go (TUG) test. Intake and exit evaluations for both VVC and F2F patient groups were performed in-person whenever possible; during periods when in-person evaluations were not possible due to COVID-19, limited evaluations (medical history, STS test) were conducted using VVC and questionnaires were sent to patients by mail.^25^ For this analysis, pulmonary diagnoses, demographics, smoking history, medications, spirometry, supplemental oxygen use, history of prior participation in PR (within 10 years), residential address, and adverse events (AEs) were extracted from the electronic medical records (EMRs). Analysis of clinical PR data was approved by the VA Boston Institutional Review Board (IRBNet No. 1578006) in 2019.

### PR Delivery Modes

Choice of delivery mode was based on shared decision-making between the patient and PR staff and prevailing infection control guidelines (see e-Appendix 1 for details). The PR program included a total of 16 to 18 sessions (two classes per week for 8 to 9 weeks), with exercise duration of 20 to 50 min per session. Education was delivered in real time by PR, pharmacy, nutrition, and behavioral health staff.

Conventional F2F PR was composed of supervised aerobic exercise training and strength training. Exercise prescriptions were targeted to achieve Borg dyspnea ratings ≤ 4 and heart rates between 60% and 80% of the maximum recorded on the 6MWT at the intake evaluation.^3^^,^^26^

The VVC-mediated synchronous PR program mirrored the center-based program except that minimal equipment was used. Patients were provided with a tablet through the VA Digital Divide initiative and a pulse oximeter, as needed. Patients exercised in their homes in groups of ≤ 10 per class. PR staff connected to VVC in the PR gym via a large monitor and led activities comprised of circuits of aerobic exercises (marching in-place, squats, lunges) and strength training (soup cans, water bottles, resistance bands); additional details are provided in e-Appendix 2. Exercise prescriptions were created in the same manner as those for the F2F program except for those who did not perform a 6MWT at baseline. Progression was guided by Borg dyspnea ratings and the age-predicted maximum heart rate (220 − age).^3^^,^^26^^,^^27^ VVC patients also received a pedometer and were encouraged to walk daily.

Since mid-February 2022, when both modalities became available, the two programs have been integrated. VVC PR is held first; after cooldown, the VVC group is joined by the F2F group for a shared education session delivered in real time. After education, the VVC group logs off and the F2F group begins warm-up and exercises.

### Outcomes

#### Safety

AEs, defined *a priori* as unexpected or worsening symptoms immediately before, during, or after class which required evaluation by medical staff, referral to urgent care/ED or dispatch of emergency medical services (for VVC patients), early termination of class, or request for additional monitoring, were captured by review of the EMR. All events were reviewed independently, in duplicate, by two pulmonologists (E. S. W. and M. L. M.) and were classified as respiratory, cardiac, musculoskeletal, or other types. Each AE was also adjudicated as PR-related or non-PR-related (eg, due to comorbidity or unrelated, intercurrent event/illness). Discordant adjudications were reviewed and discussed until agreement was achieved.

#### Feasibility/Acceptability

The number of PR classes completed for each enrollment and the proportion of enrollments where ≥ 70% of classes were completed were recorded.^28^ All patients were asked to complete a feedback questionnaire at the exit evaluation. Both groups were asked (yes/no) if they were satisfied with the program and if they would recommend the program to others. The virtual group was asked additional questions related to connectivity, clarity, and subjective sense of safety with the virtual platform.

#### Geographic Catchment

Distance and drive time from the patient’s residential address to the PR center were estimated using Google Maps’ predictive travel time set to a standardized date and time (October 18, 2022, arrive by 9 am). If multiple routes were provided, the fastest route was selected.

#### Secondary (Exploratory) Outcomes

At the intake and exit evaluations, a 6MWT was conducted according to European Respiratory Society/American Thoracic Society^29^ guidelines, except a practice test was not performed. The STS test counts the number of times a patient can stand from the seated position without the use of their hands; higher numbers indicate greater lower body strength.^30^^,^^31^ The TUG test assessed the time (in seconds) for a patient to rise from the seated position, walk 10 ft, and return to the seated position; lower numbers indicate better functional mobility.^32^ Dyspnea was self-reported using the modified Medical Research Council (range, 0-4) questionnaire, where higher numbers reflect greater dyspnea.^33^

### Statistical Analyses and Data Visualization

If a patient enrolled in more than one course of PR, each enrollment was counted as a separate observation to properly account for time-at-risk for AEs, PR completion rates, and burden of travel (primary outcomes). For secondary outcomes (change in dyspnea and functional outcomes), data from the first enrollment only was used. Subgroup analyses examined change in functional outcomes in the subset who completed ≥ 70% of PR classes.

For between-group comparisons and exploratory assessment of pre/post changes in functional outcomes, the χ^2^ or Fisher exact test (categorical) or unpaired *t* test or Wilcoxon rank sum (continuous) test was performed. Within-group changes in outcomes were assessed using paired *t* tests. Analyses were performed using R (version 4.2.1); a two-sided *P* value < .05 was considered significant. Geographic location represented by residential zip code was plotted using ArcGIS Pro (v3.1.0; Esri, Redlands, California).

## Results

Between July 30, 2020, and June 30, 2023, 174 referrals to the VA Boston PR program were received, of whom 120 observations (107 unique individuals) from patients enrolled in F2F (n = 52) or VVC (n = 68) PR and who attended one or more exercise class were included in the analysis for primary outcomes (e-Fig 1). Thirteen (seven F2F and six VVC) individuals enrolled twice in PR during the observation period; the same modality was chosen for both enrollments. Seventeen (27.4%) of 62 unique VVC patients were provided a tablet and/or internet services through VA Digital Divide.

Most patients (84%) had COPD as their primary or secondary diagnosis; among those without COPD, the most common diagnosis was interstitial lung disease (10%). The two groups were similar with respect to mean age, FEV_1_ and FVC % predicted, baseline dyspnea, and 6MWT distance (Table 1). The F2F PR group had more individuals who actively used tobacco and higher average pack-years relative to the VVC group. A greater proportion of patients in F2F PR used supplemental oxygen (34.6% vs 8.8% in VVC, *P* = .001). At baseline, the F2F group achieved a higher average number of repetitions on the STS test and lower average time on the TUG test compared with the VVC group (Table 1).

**Table 1:** Baseline Characteristics of F2F and VVC Pulmonary Rehabilitation Patients (All Enrollments)

Characteristic	F2F (n = 52)	VVC (n = 68)	Between Group *P* Value
Age, y	72.8 ± 6.8	72.7 ± 7.3	.98
Male sex	50 (96.2)	66 (97.1)	1.0
BMI, kg/m^2^	28.1 ± 6.9	29.4 ± 6.2	.31
COPD diagnosis	47 (90.4)	54 (79.4)	.17
GOLD spirometric grade (among those with COPD diagnosis and spirometry available; F2F: n = 46, VVC: n = 53)	...	...	.43
I	7 (15.2)	13 (24.5)	...
II	20 (43.5)	18 (34.0)	...
III	15 (32.6)	14 (26.4)	...
IV	4 (8.7)	8 (15.1)	...
Smoking status	...	...	...
Never	0 (0)	12 (17.6)	...
Former	42 (80.8)	52 (76.5)	...
Current	10 (19.2)	4 (5.9)	...
Smoking history, pack-years	48.5 (40.0-60.0)	40.0 (18.0-60.0)	...
FEV_1_, L (F2F: n = 51, VVC: 64)	1.72 ± 0.63	1.82 ± 0.81	.46
FEV_1_ % predicted (F2F: n = 51, VVC: n = 66)	59.4 ± 22.6	61.0 ± 26.3	.73
FVC, L (F2F: n = 51, VVC: n = 64)	3.19 ± 0.83	3.24 ± 0.87	.76
FVC % predicted (F2F: n = 51, VVC: n = 66)	82.9 ± 20.8	80.9 ± 19.5	.60
Short-acting bronchodilator use	47 (90.4)	54 (79.4)	.17
Long-acting beta-agonist use	42 (80.8)	51 (75.0)	.6
Long-acting antimuscarinic use	43 (82.7)	48 (70.6)	.19
Inhaled corticosteroid use	35 (67.3)	38 (55.9)	.28
Home oxygen use	18 (34.6)	6 (8.8)	...
mMRC (F2F: n = 50, VVC: n = 53)	1.92 ± 1.26	2.11 ± 1.03	.4
6-min walk test distance, m (F2F: n = 52, VVC: n = 34)	308 ± 102.9	277.6 ± 103.6	.19
30-s sit to stand, repetitions (F2F: n = 52, VVC: n = 65)	10.0 ± 3.5	8.1 ± 4.2	...
Timed Up and Go, s (F2F: n = 52, VVC: n = 32)	10.9 ± 3	14.0 ± 5.8	...
Previously attended pulmonary rehabilitation (within 10 y)	19 (36.5)	17 (25.0)	.24
No. of classes competed	12.4 ± 6.2	13.0 ± 6.1	.56
Driving distance, miles	10.3 (5.6-20.6)	34.1 (16.6-45.1)	...
Driving time, min	51.4 ± 31.9	86.0 ± 31.6	...

Values are as No. (%), mean ± SD, median (interquartile range), or as otherwise indicated. COPD diagnosis was considered present if listed as either the primary or secondary diagnosis for pulmonary rehabilitation. F2F = face-to-face (in-person); GOLD = Global Initiative for Chronic Obstructive Lung Disease; mMRC = modified Medical Research Council; VVC = VA Virtual Connect.

### Safety and AEs

There were 42 AEs (25 F2F and 17 VVC), of which 17 were respiratory, 10 cardiac, eight musculoskeletal, and seven other types, over 7,353 person-days of observation (Table 2). Of these, nine AEs were adjudicated to be PR-related (five F2F and four VVC); additional details are provided in e-Appendix 3. The overall rate of AEs was 5.7 events per 1,000 person-days of observation; the rate of PR-related AEs was 1.2 per 1,000 person-days of observation (Table 2). There were no significant between-group differences with respect to the average observation time per person, total AE rate, or PR-related AE rate.

**Table 2:** Cumulative AEs by PR Modality

PR Mode	AE Type				Total AEs	Observation Time		Event Rate Per 1,000 Person-Days (95% CI)	
	Respiratory	Cardiac	MSK	Other		Cumulative (days)	Average (days/person)	Total AEs	PR-Related AEs
F2F	11	8 (3 PR-related)	5 (2 PR-related)	1	25 (5 PR-related)	3,291	63.3	7.6 (4.9-11.2)	1.5 (0.5-3.5)
VVC	6 (1 PR-related)	2	3 (2 PR-related)	6 (1 PR-related)	17 (4 PR-related)	4,062	59.7	4.2 (2.4-6.7)	1 (0.3-2.5)

There were no significant differences between the event rates for total AEs and PR-related AEs by PR modality. AE = adverse event; F2F = face-to-face (in-person); MSK = musculoskeletal; PR = pulmonary rehabilitation; VVC = VA Virtual Connect.

#### Feasibility/Acceptability/Completion Rates

Among all enrollments (N = 120), the average number of exercise classes completed was comparable between F2F and VVC groups (12.4 ± 6.2 vs 13.0 ± 6.1, respectively; *P* = .56). Thirty-two (61.5%) of F2F and 46 (67.6%) of VVC enrollments completed ≥ 70% exercise classes (*P* = .62).^28^^,^^34^ Sixty-five patients completed feedback questionnaires (26 F2F and 39 VVC). All patients were satisfied with their PR experience and 98% would recommend PR to others (Table 3). Among those who responded to VVC-specific questions (n = 30), 100% reported exercise classes were easy to understand and felt safe exercising at home during the VVC sessions. Responses regarding patient experiences related to technical aspects of the VVC PR program were similarly positive (Table 3). Results were similar when repeat enrollments were excluded (data not shown).

**Table 3:** Feedback Questionnaire Responses (Exit Evaluation)

Questionnaire Item	F2F	VVC
Were you satisfied with the program? (F2F: n = 26, VVC: n = 39)	100%	100%
Would you recommend the program to others? (F2F: n = 26, VVC: n = 39)	100%	97%
The exercise classes were easy to understand. (VVC: n = 30)^a^	NA	100%
I felt safe exercising in my home during the video sessions. (VVC: n = 30)^a^	NA	100%
It was easy to connect to the program. (VVC: n = 30)^a^	NA	93%
The connection was clear. (VVC: n = 30)^a^	NA	97%
The education sessions were easy to understand. (VVC: n = 30)^a^	NA	90%

Responses are shown as % responding in the affirmative to the self-administered questionnaire item. F2F = face-to-face (in-person); NA = not applicable; VVC = VA Virtual Connect.

a VVC-only items.

#### Geographic Catchment

**Figure 1:**
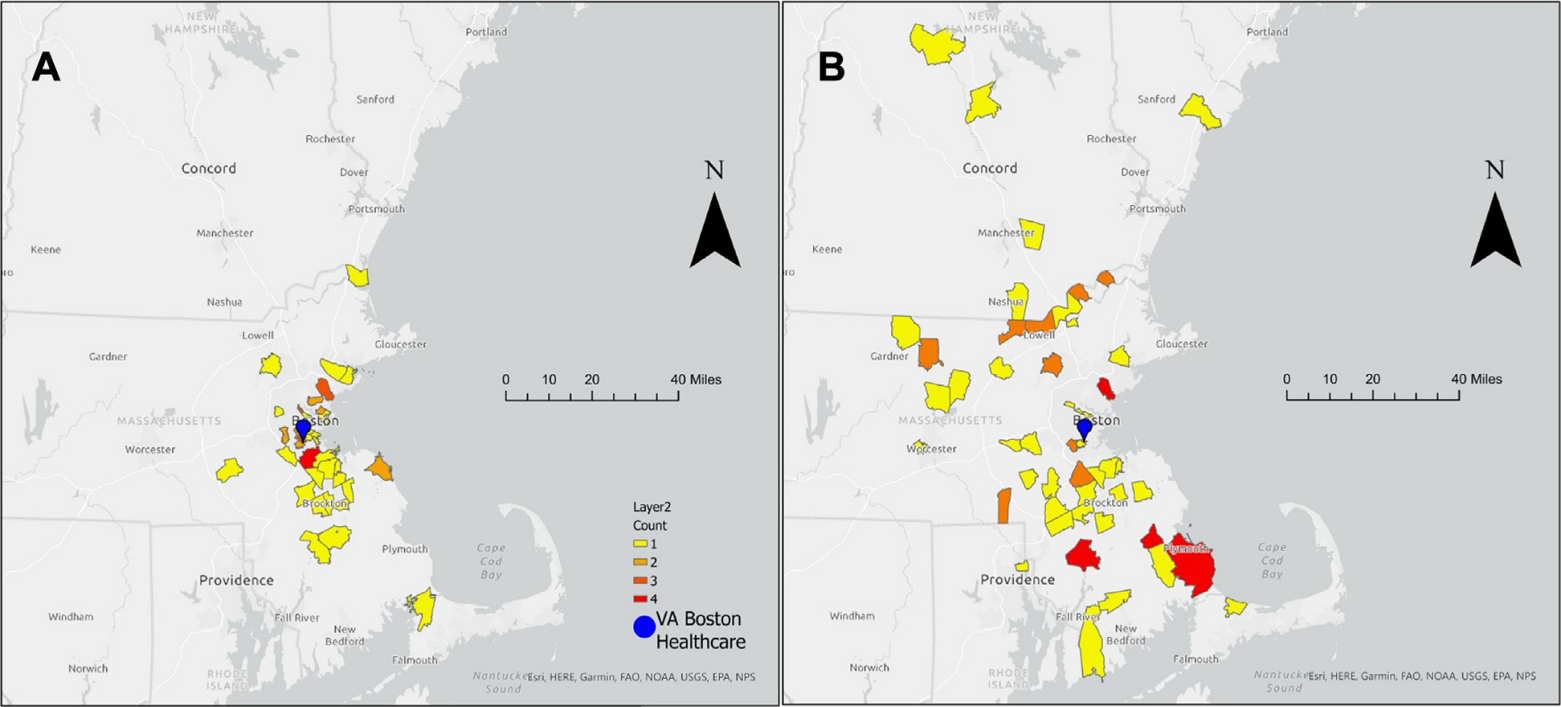
A, Geographic catchment of conventional face-to-face (n = 52) vs (B) VA Virtual Connect (n = 68) pulmonary rehabilitation programs. Zip codes of patients’ primary place of residence were used to generate maps using ArcGIS.

Patients who attended VVC PR lived significantly farther (median, 34.1; interquartile range, 16.6-45.1 vs median, 10.3; interquartile range, 5.6-20.6 miles; *P* < .001) and had longer average drive times (86.0 ± 31.6 vs 51.4 ± 31.9 min, *P* < .001) to the PR center, compared with the F2F group (Fig 1, Table 1). Results were similar when repeat enrollments were excluded (e-Fig 2, e-Table 1).

#### Functional Outcomes (Secondary Outcomes)

For the analysis of secondary exploratory outcomes, repeat enrollments (n = 13) were excluded (e-Fig 1); baseline characteristics for unique patients (n = 107) at their first enrollment are shown in e-Table 1. There were no significant differences in age, FEV_1_ % predicted, drive time or distance, or baseline functional measure between those missing vs not missing outcomes data (e-Table 2); a lower proportion of patients enrolled in VVC had change in 6MWT values available.

Both the F2F and VVC groups demonstrated significant within-group improvements in 6MWT (23.5 ± 42.2 and 40.5 ± 37.4 m, respectively) and STS test (3.1 ± 2.7 and 2.9 ± 2.5 repetitions, respectively) (Table 4). Comparable proportions of individuals (44.4% VVC and 45.8% F2F) achieved the minimal clinically important difference of 30 m for change in 6MWT.^29^ Similarly, 68.4% of VVC and 61.1% of F2F participants met the minimal clinically important difference for improvement of two repetitions in the STS test.^31^ F2F patients had a statistically significant improvement in the TUG test, whereas VVC patients demonstrated a significant improvement in modified Medical Research Council dyspnea score (Table 4). There were no significant between-group differences for changes in functional outcomes. Subgroup analyses of change in functional outcomes among the subset who completed ≥ 70% of PR classes (excluding repeat enrollments) were nearly identical (data not shown).

**Table 4:** Change in Secondary Functional Outcomes From Intake to Exit Evaluations by Pulmonary Rehabilitation Modality (Excluding Repeat Enrollments)

Outcome Measure	F2F	VVC	Between-Group *P* Value
Change in 6-min walk test distance, m (F2F: n = 24; VVC: n = 18)	23.5 (5.7 to 41.3)^a^	40.5 (21.9 to 59.1)^a^	.18
Change in 30-s sit to stand, repetitions (F2F: n = 18; VVC: n = 19)	3.1 (1.7 to 4.5)^a^	2.9 (1.8 to 4.1)^a^	.85
Change in Timed Up and Go, s (F2F: n = 18; VVC: n = 17)	−1.4 (−2.2 to −0.7)^a^	−1.8 (−3.9 to 0.4)	.75
Change in mMRC dyspnea score (F2F: n = 27; VVC: n = 31)	−0.04 (−0.51 to 0.43)	−0.48 (−0.78 to −0.19)^a^	.10

Data are shown as mean (95% CI) or as otherwise indicated. Sample sizes for each outcome vary due to missing data (eg, patient did not return for exit evaluation or did not return mMRC questionnaire). F2F = face-to-face (in-person); mMRC = modified Medical Research Council; VVC = VA Virtual Connect.

a Denotes significant within-group change from baseline (ie, average change is significantly different from zero).

## Discussion

Virtual PR and telerehabilitation are emerging models for PR delivery that are considered alternatives to, but not replacements for, center-based PR.^2^ Telerehabilitation is currently not reimbursed by most insurances in the United States due, in part, to the lack of evidence based on domestic populations.^14^ We address this gap by demonstrating that a directly supervised two-way audiovisual-teleconferenced PR program, which retains key elements of center-based programs, is safe and feasible/acceptable to patients and significantly expands geographic catchment. In exploratory analyses of efficacy, virtual PR was associated with improvements comparable with those observed in center-based PR.

Geography is a clear determinant of participation in PR, with decreased participation among those with higher travel burdens.^11^^,^^12^ Several telerehabilitation models have been explored to address this barrier. Our virtual PR program, where patients exercised in their own homes, is distinct from the hub-and-spoke model whereby hub PR programs broadcast to satellite spoke facilities where in-person, group-based PR is provided.^35^, ^36^, ^37^ Studies from the United Kingdom and Canada support comparable gains in exercise tolerance and HRQoL, along with reduced travel burden, for those traveling to spoke sites.^35^, ^36^, ^37^ Additional comparisons between hub-and-spoke models relative to in-home models, with respect to barriers and efficacy, will likely be informative. It is also notable that, although patients enrolled in our VVC program lived farther, on average, than enrollees in F2F, the range of distances traveled was wide and overlapped, with some VVC enrollees living as close as 2.3 miles and some F2F patients traveling > 50 miles. These data support that virtual programs may address barriers to PR in domains other than travel burden alone (eg, a patient’s desire to limit exposure to potential infection).^23^^,^^24^

Our study supports that virtual PR is both safe and accepted by patients. In addition to subjective reports of feeling safe while participating in virtual PR, objective rates of AEs were low and did not differ from center-based PR rates. In this real-world study, without randomization or matching, patients in both groups were similar with respect to age, lung function, dyspnea, and baseline exercise tolerance; this is distinct from a prior preference-based study where younger employed individuals were more likely to elect PR delivery via mobile devices (rather than center-based PR).^23^ It is notable that during periods when both F2F and VVC PR were available, 45% of enrollments elected the VVC option; this supports the sustained popularity of the modality among the patients, especially among the subset with high travel burden. Patients in virtual PR were also comparably engaged and had similar completion rates as those in in-person PR, supporting the overall feasibility of our virtual PR intervention.

Results from research trials, based almost exclusively among COPD populations, examining the efficacy of telerehabilitation on exercise tolerance have differed depending on the reference group used for comparison.^16^ Real-time, home-based video telerehabilitation has been reported to be comparable with in-center PR^34^ and superior to usual care (no PR)^38^ with respect to exercise tolerance/endurance. To our knowledge, there is only one other publication of a home-based video telehealth PR program in the United States using direct supervision via synchronous, two-way communication.^39^ In that study, 32 patients with COPD who were unable to enroll in center-based PR and completed video telehealth PR were retrospectively matched with 96 historical control patients who completed center-based PR. Similar to the current study, the telehealth PR intervention used minimal equipment; comparable improvements in 6MWT distance were observed in the telehealth arm relative to those seen in the historical control group.^39^ Our study extends this work to a real-world setting, with contemporaneous comparisons between virtual and center-based groups.

A key challenge for telerehabilitation is the ability to retain the essential components of center-based PR.^2^^,^^22^ At our center, comprehensive assessments of hypoxemia, comorbidities, exercise tolerance, and patient goals were key aspects of both F2F and VVC PR modalities. Because our VVC PR program used minimal equipment, the equivalence of exercise intensity and dosage to that delivered in center-based PR is unclear. Our finding that virtual PR was associated with similar improvements in dyspnea and exercise tolerance relative to center-based PR may support that the relative training loads were effective in each group. In a telerehabilitation model using minimal resources including water bottles for strength training, a trend toward greater improvement in 6MWT was observed relative to center-based PR,^40^ whereas another program that used repetitive sets of time-based muscle endurance training demonstrated a trend toward higher gains in the center-based program.^34^ A recent meta-analysis demonstrated that, among people with COPD, PR programs using minimal equipment elicited improvements in 6MWT distance comparable with programs with exercise equipment.^41^

We acknowledge several limitations. First, due to COVID-19, evolving social distancing and masking requirements during our reporting period may have unclear impacts on our results. Self-selection bias may contribute to nonrandom missingness for functional outcome measurements (eg, individuals who completed an exit evaluation may have improved the most). Notably, baseline characteristics between those missing and not missing data on secondary functional outcomes data were similar (e-Table 2). Our study was not powered to detect between-group differences in the functional outcome measures; an independent, multisite pragmatic (practical) trial, including assessments of change in HRQoL, may be warranted to confirm these outcomes. Second, given that the cohort was predominantly male with moderate impairment on spirometry, generalizability to other populations may be limited. In addition, the smaller proportion of individuals requiring supplemental oxygen in the VVC arm may also limit generalizability; guidelines regarding eligibility for virtual PR are needed. Third, our feedback questionnaire was composed of binary responses and has not been externally validated. Although this questionnaire was self-administered, unconscious pressure to answer positively among respondents cannot be ruled out. Fourth, anonymization to PR modality during the extraction of AE data from the EMR was not possible; to minimize the potential impact, all AE assessments were performed independently in duplicate using well-defined criteria supported by medical documentation. Whether systematic differences in observation time or the threshold to report symptoms or events to PR providers between modalities exist is also unknown; future reports from independent programs will be informative. Fifth, although VVC PR retained the core elements of aerobic and strength training, exercise dosage (or volume) was not strictly measured and varied over time as the program evolved. Despite this, our exploratory analyses of functional outcomes support that VVC PR was effective based on significant within-group gains. Finally, although a pedometer was used in VVC PR to encourage physical activity outside of class, our virtual PR intervention is not a physical activity promotion program; the effect of this component of the program remains incompletely assessed. Despite these limitations, we assert our real-world report comparing center-based and virtual PR, both during and after periods of unprecedented disruptions due to COVID-19, supports that the addition and uptake of virtual PR is both safe and feasible. Real-time, two-way audiovisual teleconferencing is a potential option to increase access to PR for patients with chronic lung disease.

## Funding/Support

This work was indirectly supported, in part, by the 10.13039/100000738US Department of Veterans Affairs, 10.13039/100006379Office of Research and Development, Rehabilitation Research and Development Career Development Award [Award RX002165 to E. S. W.] and 10.13039/100015728Clinical Science Research and Development Merit Award [Award CX002193 to E. S. W.].

## Financial/Nonfinancial Disclosures

The authors have reported to *CHEST Pulmonary* the following: E. S. W. has served on a scientific advisory board for Verona Pharmaceuticals, outside the current work. None declared (J. D., C. S., J. R. V., B. M., S. A. R., P. B., M. L. M.).

## Supplementary Data

e-Online Data
