# Supplementary material for: Safety and Feasibility of a Two-Way Audiovisual Teleconferenced Pulmonary Rehabilitation Program
Source: CHEST Pulm. 2024 Jul 29;3(2):100089. doi: 10.1016/j.chpulm.2024.100089 (PMC12716338; doi:10.1016/j.chpulm.2024.100089)
Supplement: e-Online Data [file mmc1.docx]

A Two-Way Audiovisual Teleconferenced Pulmonary Rehabilitation Program Is

Safe, Feasible, and Expands Geographic Catchment

Emily S. Wan, MD, MPH, Josephine Decherd, MPH, Christine Stella, NP,

Jonathan R. Venne, PT, Brenda McKeon, NP, Stephanie A. Robinson, PhD,

Patricia Bamonti, PhD, Marilyn L. Moy, MD, MSc

**SUPPLEMENTAL DATA**

**Supplemental Methods**

PR Delivery Modes

The VA Boston PR Program is a half-time program located in the Jamaica Plain neighborhood of Boston, Massachusetts which is staffed by a pulmonologist, nurse practitioner/physician assistant, physical therapist/kinesiotherapist (KT), and respiratory therapist. In spring 2020, the Veterans Health Administration promoted the use of VA Virtual Connect (VVC), a secure, HIPAA-compliant platform, to deliver clinical care. During the observation period for this analysis, the availability of face-to-face (F2F) PR varied based on local COVID-19 social distancing restrictions. VVC as a delivery mode option for PR was available for the entire period under consideration, with the first virtual intake evaluation occurring on 7/30/20 and the first virtual exercise class delivered on 8/18/2020. VVC PR was the only option available between August, 2020 and May, 2021. In May, 2021, limited F2F PR resumed, but from January 1, 2022 to February 8, 2022, VVC was the only PR option since F2F classes were closed due to a COVID-19 surge in the community. Since mid-February, 2022, both F2F and VVC options have been available. During periods when both PR modalities were available, the delivery mode chosen for each enrollment was decided on a case-by-case basis using shared-decision making, accounting for safety and the patient’s personal preferences. Patients were provided with descriptions of both F2F and VVC options by PR providers at the point of referral and again at the time of intake evaluation.

*In-person face-to-face (F2F) PR program*

Conventional center-based PR was delivered in-person. PR staff and patients wore a mask during the classes when local guidance required it. Patients could have a “mask break” at any time. COVID-19 antigen testing prior to participation in F2F PR was performed per local guidelines in effect at the time. Direct in-person supervision was provided by PR staff. The in-person F2F group joined the VVC group for education delivered by in-person staff in the gym. In-person education was provided by a multi-disciplinary team from PR, pharmacy, nutrition, and behavioral psychology. After education was complete, the F2F group began warm-ups, strength training with resistance bands or free weights, aerobic exercise training on the gym equipment, and cool-down. Exercise equipment in the gym included: treadmills, recumbent and upright bikes, recumbent steppers, arm ergometers, free weights, and resistance bands.

*Virtual VVC PR Program*

The VVC-mediated, synchronous, real-time PR program delivered via 2-way audiovisual teleconferencing was developed by the medical director and a KT in July, 2020. Patients were requested to attend an in-person intake and exit evaluation, which included a 6MWT, whenever possible; when an in-person intake was not possible, a virtual intake was performed. Exercise prescriptions were created and implemented in the same way as those for the F2F program. Progression of exercise targeted Borg ratings for dyspnea of no higher than 4 and heart rates between 60-80% of the maximum recorded at the end of the intake 6MWT. For those who did not perform a 6MWT at baseline, progression was guided by Borg ratings for dyspnea, with knowledge of the age-predicted maximum heart rate.

Patients exercised in their homes, in groups of no more than 10 on the monitor with real-time communication enabled via the VVC platform. PR staff performed exercises in the gym in front of the monitor with the patients following along in their homes. PR staff ensured that everyone’s entire body was visible on the monitor, provided real-time feedback on execution and safety, and monitored patient-reported heart rate and oxygen saturation data. Similar to the F2F program, exertional hypoxemia was managed acutely with rest and monitoring, if symptomatic. Titration of a patient’s oxygen prescription (for example, increasing oxygen flow rate) or use of reservoir systems (e.g., nasal pendant) under the guidance of the PR respiratory therapist was also offered for recurrent exertional hypoxemia. Oxygen prescriptions initiated or modified during PR were then communicated to the home oxygen supplier and the patient’s medical team.

Exercise, targeted to individual Borg dyspnea ratings and HR, was comprised of warm-up, static stretches, active movement with sit-to-stands, squats, and marching in place, and strength exercises with 1-2-pound water bottles soup cans, or resistance bands, followed by cool-down. After the KT retired and a physical therapist started in November, 2021, the aerobic exercise routine was modified to include 3 sets of a circuit of 10 exercises (i.e., marching in place – simple and opposite knee, front, side, and reverse step lunges, mini-squats alternating punches, shuffle steps) – please see sample exercise circuit (Appendix 1). Strength training was followed by a cool-down period as outlined above (Appendix 1). Virtual PR patients then shared the same live education sessions with the F2F group.

The VVC program also included an unsupervised walking component. Patients were instructed to walk every day, using the Borg scale to monitor dyspnea and a pedometer to track daily step counts and heart rate. Weekly step-count data were self-reported and reviewed by PR staff. If patients were clinically stable, they were provided a new goal for the upcoming week with 400 steps per day added to the average daily step count from the previous week^1,2^.

*VA Telehealth Infrastructure and Support*

Veterans without an internet-capable device were loaned a tablet through the VA Digital Divide initiative (https://telehealth.va.gov/digital-divide). The service also assesses and assists with internet service and data charges for eligible Veterans. Following delivery of the device and/or establishment of internet service, the VA Telehealth Support team assists patients with the setup of the loaner device and can conduct a test call via the VVC system. In addition, all healthcare providers who utilize the VVC platform are required to complete standardized trainings on the management of emergencies. At the beginning of each VVC session, emergency contact numbers for a patient’s location are acquired and verified. During an unexpected AE, a healthcare provider maintains contact either via the VVC platform or via the backup telephone number until event resolution or on-site support has arrived.

*Functional Outcomes Assessments*

The 30-second sit-to-stand test (STS)^3,4^ and timed-up-and-go (TUG)^5^ test were conducted according to the U.S. Centers for Disease Control (CDC) guidelines. Additional details are available at: https://www.cdc.gov/steadi/pdf/STEADI-Assessment-30Sec-508.pdf (30-second STS; accessed 2/15/2024) and https://www.cdc.gov/steadi/pdf/TUG_test-print.pdf (TUG; accessed 2/15/2024).

# WARM UP

1. Shake out your arms
2. Neck circles (pain free motion only, 3-4 circles in each direction)
3. Tip your ear to your shoulder (contralateral neck stretch; perform on both sides, repeat 3-4 times)
4. Shoulder rolls (3-4 in each direction)
5. Low back side and back bends (left, right, and backward; no pain, repeat 3 times)
6. Trunk twist (3-4 in each direction)
7. Swimming (forward crawl and backstroke; 5 times in each direction)
8. Calf stretch (20-30 on seconds each side)

# AEROBIC CIRCUIT TRAINING (VVC)

10 Exercises, 30 second intervals, 3 Circuits, 15 Minutes

1. Marching
2. Heel Tap/Forward Lunge
3. Marching, Opposite Hand/Opposite Knee
4. Side-step/Side Lunge Left
5. Side-step/Side Lunge Right
6. Marching w/ Hand Claps
7. Toe Tap/Reverse Lunge
8. Marching
9. Mini-squat w/ Alternating Punch
10. Step-Left-Middle-Right/Shuffle Step

# STRENGTH

- Can be performed seated or standing
- 2 seats, 8-12 repetitions of each
- Sit-to-stands between each pair
- RPD = Rating of Perceived Dyspnea

| **Shoulder Front Raise (light)** | 8-12 Repetitions |
| --- | --- |
| **Bent Over Row (heavy)** | 8-12 Repetitions |
| **Sit-to-stand (as tolerated, RPD <4)** | **30 Seconds** |
| **Shoulder Front Raise (light)** | 8-12 Repetitions |
| **Bent Over Row (heavy)** | 8-12 Repetitions |
| **Sit-to-stand (as tolerated, RPD <4)** | **30 Seconds** |
| **Triceps Kickback (light)** | 8-12 Repetitions |
| **Biceps Curl (heavy)** | 8-12 Repetitions |
| **Sit-to-stand (as tolerated, RPD <4)** | **30 Seconds** |
| **Triceps Kickback (light)** | 8-12 Repetitions |
| **Biceps Curl (heavy)** | 8-12 Repetitions |
| **Sit-to-stand (as tolerated, RPD <4)** | **30 Seconds** |
| **Overhead Press (light or heavy)** | 8-12 Repetitions |
| **Wall Push-ups** | 8-12 Repetitions |
| **Sit-to-stand (as tolerated, RPD <4)** | **30 Seconds** |
| **Overhead Press (light or heavy)** | 8-12 Repetitions |
| **Wall Push-ups** | 8-12 Repetitions |

# STANDING BALANCE

- Performed in standing
- Performed at each person’s own pace (no out loud counting)
- Task difficulty increases as hand support decreases, so patients are encouraged to hold on only as much as necessary for safety (and reduce over time as they improve)

| **Heel Raise** | Both Feet: 10-20 Reps  One Foot: 5-10 Reps each |
| --- | --- |
| **Hip Abduction (side lift)** | 10 Reps/15 sec |
| **Hip Flexion (front lift)** | 10 Reps/15 sec |
| **Hip Extension (back lift)** | 10 Reps/15 sec |

# COOLDOWN/STRETCHES

| **Standing Calf Stretch** | 15-20 sec (each side) |
| --- | --- |
| **Seated Hamstring Stretch** | 15-20 sec (each side) |
| **Figure 4 Hip Stretch** | 15-20 sec (each side) |
| **Reach Across Shoulder Stretch** | 15-20 sec (each side) |
| **Finger Flexor Stretch** | 15-20 sec |

**Supplementa1 Results**

*Primary versus Secondary COPD diagnoses*

Of the 101 observations (84% of total cohort) with COPD, most (n=96, 95%) had COPD listed as the primary diagnosis. Of the remaining 5 patients where COPD was technically listed as the secondary diagnosis, 2 were pre-transplant (for COPD), 1 patient had asthma-COPD overlap, 1 patient had combined ILD and COPD, and 1 patient had COVID-19 superimposed on pre-existing COPD.

*Safety and Adverse Events*

Patients with a diagnosis of COPD accounted for 88% of those with respiratory AEs, 90% of those with cardiac AEs, 100% of those with MSK AEs and 71% of those with AEs classified as “Other”. These results support a likely high rate of comorbid conditions among individuals with COPD.

*PR-related AE events – F2F*

Of the 5 PR-related AEs in the F2F group, 3 events were cardiac in nature: 1 patient experienced extreme hypertension during exercise (resolved with rest; care coordination with primary care to titrate antihypertensive medications), 1 patient developed hemodynamically-stable wide-complex tachycardia during exercise (referred to urgent care, subsequently admitted and started on nodal blockade), 1 patient developed substernal chest pain while on the treadmill (taken to urgent care, subsequently admitted and required cardiac stent placement).

Of the 2 musculoskeletal events adjudicated to be PR-related, 1 patient experienced bilateral hip pain while exercising on a recumbent bicycle and 1 patient developed worsening lower back pain. Both patients were conservatively managed (ice, non-prescription analgesics) and recovered by the next session.

*PR-related AE events – VVC*

There were 4 PR-related AEs in the VVC group. One patient experienced a respiratory AE characterized by severe hypoxemia (SpO_2_<85%) with exercise which was refractory to increasing his home oxygen (resolved with rest and pursed lip breathing; later additionally addressed by providing a nasal pendant and increased flow rates with exercise). Of the 2 musculoskeletal PR-related AEs, one was due to neck pain during resistance training (resolved with rest, non-prescription analgesics; addressed by modification of resistance training maneuvers/technique) and one was due to worsening lower back pain (resolved with rest and non-prescription medications). The PR-related event classified as “Other” was due to subjective lightheadedness and dizziness during exercise despite reassuring vital signs (managed conservatively, able to participate in subsequent classes without recurrence).

**Supplementary Figures**

**e-Figure 1**

Referred to Pulmonary Rehabilitation Program and completed an intake evaluation (N=145)

Attended ≥ 1 Pulmonary Rehabilitation exercise class (N=123)

Attended Pulmonary Rehabilitation

exercise classes using either F2F or VVC

(**N=120; 52 F2F and 68 VVC**)

Attended Pulmonary Rehabilitation exercise classes in both Face-to-Face (F2F)

and VA Video Connect (VVC) modalities (N=3)

Did not attend any Pulmonary Rehabilitation exercise classes (N=22)

Referred to Pulmonary Rehabilitation Program between 7/30/2020-6/30/2023 (N=174)

Not interested or did not complete an intake evaluation (N=29)

Unique patients (first enrollment only)

(**N=107; 45 F2F and 62 VVC**)

Repeat enrollments removed prior to assessment of change in functional outcomes (N=13; 7 F2F and 6 VVC)

**Primary Outcomes Analysis Population**

Secondary Outcomes Analysis Population

**e-Figure 2** – Geographic catchment of conventional face-to-face (F2F; N=45; panel A) versus VA Virtual Connect (VVC; N=62; panel B) Pulmonary Rehabilitation programs excluding repeat enrollments. Zip codes of patients’ primary place of residence were used to generate maps using ArcGIS.


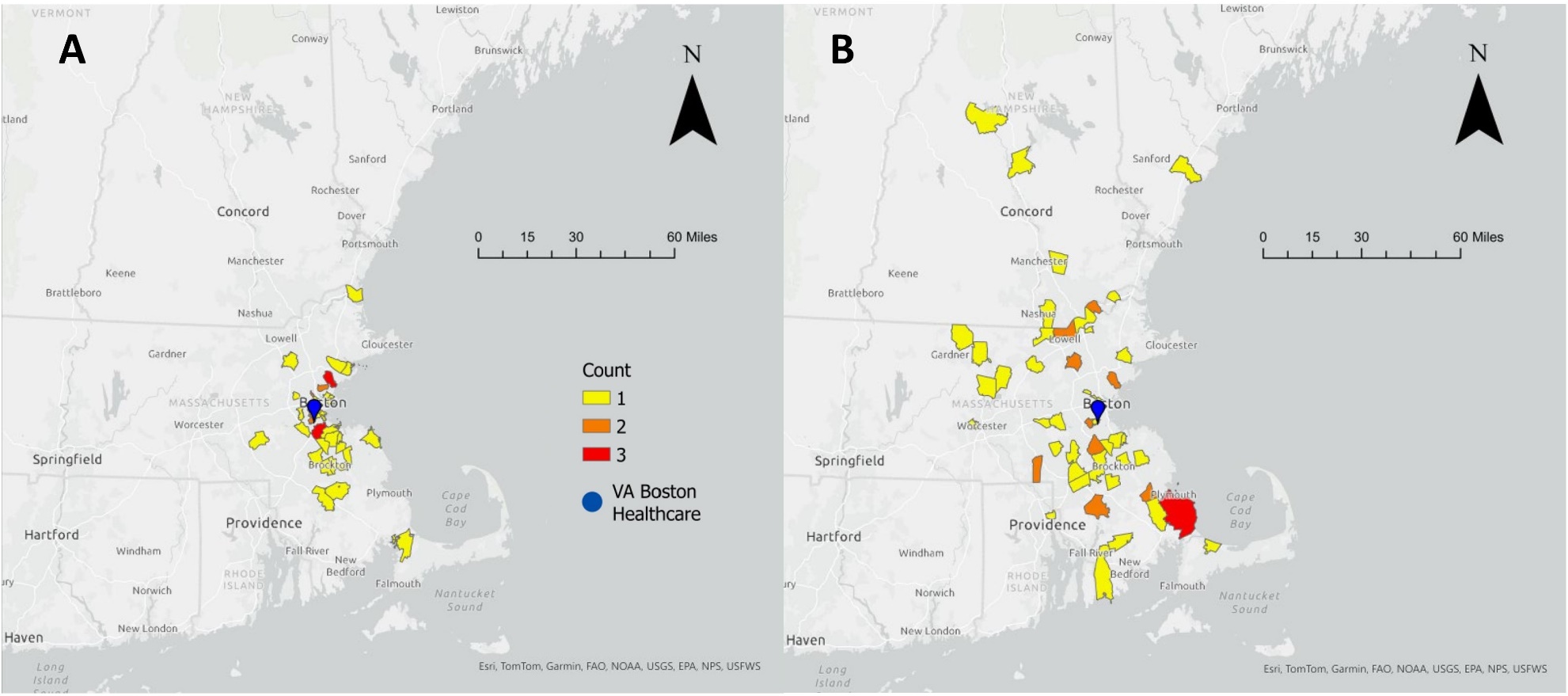


**Supplemental Tables**

**e-Table 1** – Baseline characteristics of unique Face-to-Face (F2F) and VA Virtual Connect (VVC) Pulmonary Rehabilitation Patients

(excluding repeat enrollments)

|  | **F2F** | **VVC** | ***P* value<0.05** |
| --- | --- | --- | --- |
| N | 45 | 62 |  |
| Age (years) | 72.4 ± 6.6 | 73 ± 7.3 |  |
| Male Sex | 43 (95.6%) | 60 (96.8%) |  |
| COPD Diagnosis | 40 (88.9%) | 49 (79.0%) |  |
| Smoking Status |  |  | * |
| Never | 0 (0%) | 12 (19.4%) |  |
| Former | 36 (80.0%) | 46 (74.2%) |  |
| Current | 9 (20.0%) | 4 (6.5%) |  |
| Smoking history (pack-years) | 47.0 [31.0, 60.0] | 40.0 [17.3, 60.0] | * |
| FEV_1_ (Liters) (N=44 F2F; 58 VVC) | 1.70 ± 0.65 | 1.84 ± 0.82 |  |
| FEV_1_ % predicted (N=44 F2F; 60 VVC) | 58.4 ± 22.8 | 61.3 ± 25.8 |  |
| FVC (Liters) (N=44 F2F; 58 VVC) | 3.16 ± 0.82 | 3.26 ± 0.90 |  |
| FVC % predicted (N=44 F2F; 60 VVC) | 81.6 ± 20.4 | 81.0 ± 19.5 |  |
| Home Oxygen Use | 16 (35.6%) | 3 (4.8%) | * |
| mMRC (N=44 F2F; 48 VVC) | 1.82 ± 1.24 | 2.15 ± 1.05 |  |
| 6-Minute Walk Test Distance (meters) (N=45 F2F; 29 VVC) | 300.5 ± 100.9 | 282.2 ± 108.5 |  |
| 30-Second Sit to Stand (repetitions) (N=45 F2F; 59 VVC) | 9.6 ± 3.4 | 8.1 ± 4.3 |  |
| Timed Up and Go (seconds) (N=45 F2F; 27 VVC) | 11 ± 3.1 | 14.1 ± 6.3 | * |
| Driving Distance (miles) | 11.7 [5.8, 20.6] | 31.4 [16.2, 44.9] | * |
| Driving Time (minutes) | 53.4 ± 31.7 | 84.7 ± 32.5 | * |
| F2F = Face-to-Face (in-person); VVC = VA Virtual Connect; FEV_1_=forced expiratory volume in the first second; FVC=forced vital capacity; mMRC = modified Medical Research Council dyspnea score.  COPD diagnosis was considered present if listed as either the primary or secondary diagnosis for Pulmonary Rehabilitation.  Values are shown as N (%), mean ± SD or median [IQR].  *Denotes *P*<0.05 for significance testing between F2F versus VVC groups. | | | |

**e-Table 2** – Differences in Baseline Characteristics by Missingness in Secondary Functional Outcome Variable (excluding repeat enrollments)

|  | Missing Change in Outcome Measure | Not Missing Change in Outcome Measure |
| --- | --- | --- |
| **6-Minute Walk Test (6MWT)** | | |
| N | 65 | 42 |
| Modality: VVC | 44 (68%) | 18 (43%)* |
| Age | 72.4 ± 7.2 | 73.2 ± 6.6 |
| FEV_1_ % predicted | 60.8 ± 26.0 | 58.9 ± 22.3 |
| Drive Distance (miles) | 21.9 [9.7, 39.4] | 20.4 [10.5, 29.7] |
| Drive Time (minutes) | 74.5 ± 36.8 | 67.0 ± 33.6 |
| Baseline 6MWT (meters) | 269 ± 122.9 | 311.9 ± 82.9 |
| **Modified Medical Research Council (mMRC) Dyspnea Score** | | |
| N | 49 | 58 |
| Modality: VVC | 31 (63.3%) | 31 (53.4%) |
| Age | 72.5 ± 7.2 | 72.9 ± 6.8 |
| FEV_1_ % predicted | 61.2 ± 26.1 | 59.2 ± 23.3 |
| Drive Distance (miles) | 18.3 [7.0, 39.0] | 22.6 [13.7, 38.2] |
| Drive Time (minutes) | 67.3 ± 35.9 | 75.1 ± 35.4 |
| Baseline mMRC Score | 2.0 ± 1.3 | 2 ± 1.1 |
| **30-second Sit to Stand (STS)** | | |
| N | 70 | 37 |
| Modality: VVC | 43 (61.4%) | 19 (51.4%) |
| Age | 72.2 ± 7.1 | 73.8 ± 6.8 |
| FEV_1_ % predicted | 60.9 ± 26.2 | 58.5 ± 21.3 |
| Drive Distance (miles) | 19.7 [9.2, 39.1] | 22.2 [11.7, 31.9] |
| Drive Time (minutes) | 71.4 ± 36. | 71.8 ± 34.3 |
| Baseline STS (number of repetitions) | 9.0 ± 4.1 | 8.4 ± 3.7 |
| **Timed Up and Go (TUG)** | | |
| N | 72 | 35 |
| Modality: VVC | 45 (62.5%) | 17 (48.6%) |
| Age | 72.7 ± 7.2 | 72.9 ± 6.5 |
| FEV_1_ % predicted | 59.6 ± 25.7 | 61.1 ± 22.1 |
| Drive Distance (miles) | 20.8 [9.7, 39.2] | 20.8 [10.6, 31.3] |
| Drive Time (minutes) | 73.5 ± 36.4 | 67.5 ± 34.2 |
| Baseline TUG (seconds) | 13.0 ± 5.7 | 11.3 ± 3.5 |
| Data are shown as N (%), mean ± SD, or median [IQR].  * Denotes between-group difference significant at *P*<0.05. | | |

**Supplementary References**

1. Robinson SA, Cooper JA, Jr., Goldstein RL, et al. A randomised trial of a web-based physical activity self-management intervention in COPD. ERJ Open Res 2021;7(3). DOI: 10.1183/23120541.00158-2021.

2. Wan ES, Kantorowski A, Homsy D, et al. Promoting physical activity in COPD: Insights from a randomized trial of a web-based intervention and pedometer use. Respir Med 2017;130:102-110. DOI: 10.1016/j.rmed.2017.07.057.

3. Jones CJ, Rikli RE, Beam WC. A 30-s chair-stand test as a measure of lower body strength in community-residing older adults. Res Q Exerc Sport 1999;70(2):113-9. DOI: 10.1080/02701367.1999.10608028.

4. Zanini A, Crisafulli E, D'Andria M, et al. Minimum Clinically Important Difference in 30-s Sit-to-Stand Test After Pulmonary Rehabilitation in Subjects With COPD. Respir Care 2019;64(10):1261-1269. DOI: 10.4187/respcare.06694.

5. Podsiadlo D, Richardson S. The timed "Up & Go": a test of basic functional mobility for frail elderly persons. J Am Geriatr Soc 1991;39(2):142-8. DOI: 10.1111/j.1532-5415.1991.tb01616.x.
